# Hypercholesterolemia in the Malaysian Cohort Participants: Genetic and Non-Genetic Risk Factors

**DOI:** 10.3390/genes14030721

**Published:** 2023-03-15

**Authors:** Nor Azian Abdul Murad, Yusuf Mohammad Noor, Zam Zureena Mohd. Rani, Siti Aishah Sulaiman, Yock Ping Chow, Noraidatulakma Abdullah, Norfazilah Ahmad, Norliza Ismail, Nazihah Abdul Jalal, Mohd. Arman Kamaruddin, Amalia Afzan Saperi, Rahman Jamal

**Affiliations:** 1 UKM Medical Molecular Biology Institute (UMBI), Universiti Kebangsaan Malaysia (UKM), Jalan Yaacob Latiff, Cheras, Kuala Lumpur 56000, Malaysia; 2 Malaysian Genome Institute (MGI), Jalan Bangi, Bangi 43000, Malaysia; 3 Department of Community Health, Faculty of Medicine, Universiti Kebangsaan Malaysia (UKM), Jalan Yaacob Latiff, Cheras, Kuala Lumpur 56000, Malaysia

**Keywords:** hypercholesterolemia, whole-exome sequencing, Malaysian Malay, genetics, clinical and environmental factors

## Abstract

Hypercholesterolemia was prevalent in 44.9% of The Malaysian Cohort participants, of which 51% were Malay. This study aimed to identify the variants involved in hypercholesterolemia among Malays and to determine the association between genetic and non-genetic risk factors. This nested case–control study included 25 Malay participants with the highest low-density lipoprotein cholesterol (LDL-C, >4.9 mmol/L) and total cholesterol (TC, >7.5 mmol/L) and 25 participants with the lowest LDL-C/TC. Genomic DNA was extracted, and whole-exome sequencing was performed using the Ion Proton^TM^ system. All variants were annotated, filtered, and cross-referenced against publicly available databases. Forty-five selected variants were genotyped in 677 TMC Malay participants using the MassARRAY^®^ System. The association between genetic and non-genetic risk factors was determined using logistic regression analysis. Age, fasting blood glucose, tobacco use, and family history of hyperlipidemia were significantly associated with hypercholesterolemia. Participants with the novel OSBPL7 (oxysterol-binding protein-like 7) c.651_652del variant had 17 times higher odds for hypercholesterolemia. Type 2 diabetes patients on medication and those with PCSK9 (proprotein convertase subtilisin/kexin type 9) rs151193009 had low odds for hypercholesterolemia. Genetic predisposition can interact with non-genetic factors to increase hypercholesterolemia risk in Malaysian Malays.

## 1. Introduction

Hypercholesterolemia is one of the risk factors for cardiovascular disease (CVD), the leading cause of mortality and morbidity worldwide [1]. Based on the 2015 Malaysian Ministry of Health report, the overall prevalence of hypercholesterolemia among Malaysian adults was 47.7%, and 38.6% were undiagnosed [2]. A recent publication from The Malaysian Cohort (TMC) project, a multi-ethnic population cohort, showed that hypercholesterolemia was prevalent in 44.9% of the participants, of which 51% were of Malay descent [3]. Hypercholesterolemia is a multi-factorial disease; the primary cause is genetic predisposition, and the secondary causes include unhealthy diet, smoking, and hypothyroidism [4].

The genetic disorder in hypercholesterolemia refers to familial hypercholesterolemia (FH), which causes elevated low-density lipoprotein cholesterol (LDL-C) levels [5]. Currently, the genes involved in FH can be divided into tier 1, 2, and 3 genes based on their involvement in lipid metabolism [5,6,7,8]. The tier 1 genes include that for LDL receptor (*LDLR*), apolipoprotein B-100 (*APOB*), and proprotein convertase subtilisin/kexin type 9 (*PCSK9*), which follow the autosomal dominant mode of inheritance, and LDLR adaptor protein 1 (*LDLRAP1*), which follows the autosomal recessive mode of inheritance (directly implicated in FH) [5,6]. Tier 2 genes are indirectly implicated in FH, but are associated with regulation of LDL or affects the expression of LDL-regulating genes, whereas tier 3 genes are the other genes implicated in lipid regulation [7,8]. FH is a genetic disease with gene dosage effects, being more severe in homozygous compared to heterozygous patients [9]. Most of the common mutations are reported in the *LDLR* gene (90%) [10]. However, the majority of patients have polygenic hypercholesterolemia, which results from the interaction of genetic factors with a sedentary lifestyle and increased intake of dietary fats [4].

In Malaysia, the frequency of heterozygous and homozygous FH is one in 500 and one in 1 million, respectively, but the actual frequency may be higher due to underdiagnosis [11]. Thus, several studies have attempted to determine the mutational profiles in Malaysian FH [12,13,14,15]. However, most of these studies are small and focus only on either the *LDLR* or *APOB* gene. Lye and colleagues investigated 1536 single-nucleotide polymorphisms (SNPs) in 141 FH patients characterized by the Dutch Lipid Clinic Network criteria, and in 111 unrelated controls, and found that 14 SNPs were significantly associated with FH (high-risk: 11, low-risk: 3) [16]. Even then, causative variants were not detected in about 23.4% of FH patients [16], indicating that FH in these patients was caused by possible unknown genetic variants. Therefore, we aimed to comprehensively identify the mutational profiles of participants with hypercholesterolemia of Malay descent from the TMC study via whole-exome sequencing (WES). Subsequently, we validated the findings via genotyping and determined the genetic and non-genetic factors involved in hypercholesterolemia in these patients.

## 2. Materials and Methods

### 2.1. Sampling, Data Collection, and Study Design

Participants were selected from the TMC project, an ongoing nationwide prospective project that has recruited 106,527 Malaysians aged 35–70 years [3]. Sociodemographic and lifestyle information were collected via questionnaires and interviews, together with collection of the relevant biophysical and biochemical measurements. The nested case–control study design was used for the discovery phase (WES). Lipid profiles (total cholesterol (TC), LDL-C, triglyceride (TG), high-density lipoprotein cholesterol (HDL-C)) were determined on a Cobas Integra 800 analyzer (Roche Diagnostics, Germany). Based on the Simon Broome criteria, the cut-off points for hypercholesterolemia were 4.9 mmol/L for LDL-C and 7.5 mmol/L for TC. The LDL-C and TC levels were ranked, and 25 participants with the highest LDL-C/TC levels (denoted as HLDL) were selected as the case group. Twenty-five participants with the lowest levels of TC (<5.2 mmol/L) and LDL-C (2.6–3.4 mmol/L) [17] (denoted as LLDL) were selected. All participants were of Malay descent and could be traced back at least three generations. The other inclusion criteria were: (i) Malaysian citizenship; (ii) age 35–70 years; (iii) without debilitating illnesses at the time of the study; (iv) provided written informed consent. The study was approved by the Universiti Kebangsaan Malaysia (UKM) Research Ethics Committee (Ethics Number: FF-205-2007). The characteristics of the 25 HLDL and 25 LLDL participants are summarized in Supplementary Tables S1 and S2, respectively.

### 2.2. DNA Isolation and WES

DNA was isolated from 200 µL whole blood using a KingFisher™ Pure DNA Blood Kit (Thermo Fisher Scientific, Waltham, MA, USA) and KingFisher™ Duo system (Thermo Fisher Scientific) according to the manufacturer’s protocols. DNA was quantified with a Qubit™ Broad-Range DNA Quantification Kit (Invitrogen, Carlsbad, CA, USA). High-quality DNA (0.5–1.5 μg) was sheared into 150–250 bp fragments using a Covaris S2 ultrasonicator (American Laboratory Trading, East Lyme, CT, USA). WES was performed using an Ion P1 200 Sequencing Kit (Thermo Fisher Scientific) according to the manufacturer’s protocol. Enriched libraries were sequenced as single-end 150 bp reads on the Ion Proton™ system (Thermo Fisher Scientific).

### 2.3. Bioinformatics Data Analysis

Torrent Suite v4.0.2 (Thermo Fisher Scientific) was used for quality control metrics, including bead loading percentage, read length, percentage of alignment to the reference hg19, and total usable sequences. Base recalibration and duplicate removal were applied to the raw data sequence. We ensured that the raw data for each sample had at least 30× mean coverage and 20× coverage for at least 70% of the targeted regions. Germline mutations were detected at 50× coverage [18]. Variants were called using the Torrent Variant Caller plugin with default parameters for Germline Proton TargetSeq Low Stringency. All variants for each sample were annotated and filtered using ANNOVAR [19]. Variants were cross-referenced against the publicly available dbSNP 138, 1000 Genomes Project (April 2012 release), and Exome Sequencing Project databases. The variant effect on the protein structure was predicted using PolyPhen-2 [20] and SIFT [21]; all variants with tolerable effects were filtered out. The variants were classified using the knownGene annotation (University of California Santa Cruz); only variants in exonic regions were considered; synonymous SNPs were filtered out, and frameshift and stop-gain/stop-loss variants were shortlisted for downstream priority over non-synonymous SNPs and non-frameshift insertions/deletions (indels). Variants were also filtered based on whether they were in the FH genes. Tier 1 gene (*LDLR*, *APOB*, *PCSK9*, *LDLRAP1*) [5,6] variants were given higher priority over tier 2 and 3 gene variants [7,8]. All variants were assessed based on frequency in the HLDL and LLDL samples. Variants exclusively present in HLDL samples were identified as causative, whereas variants present only in the LLDL samples were classified as protective. The impact of these variants was assessed using in silico prediction tools: SIFT [21], PolyPhen-2 [20], MutationTaster [22], and FATHMM [23]. The clinical significance of all known variants was confirmed based on the ClinVar database (https://www.ncbi.nlm.nih.gov/clinvar (accessed on 23rd June 2021)). The WES data generated have been submitted to the National Center Biotechnology Information (NCBI) Sequence Read Archive (SRA) under accession number PRJNA607111.

### 2.4. Internal Validation of 45 Variants

In total, 27 variants that increased risk (Supplementary Table S3) and 18 variants that potentially reduced risk (Supplementary Table S4) were selected for replication in a larger sample size. Sample size was calculated assuming an additive model, perfect linkage disequilibrium between risk and marker, 80% power of study, and 47.7% prevalence of hypercholesterolemia [3]. For a genetic risk ratio of 14.78 (*APOB* rs12720762, minimum allele frequency [MAF] = 0.0056), the sample size needed was 22, while for genetic risk ratio of 1.77 (*LDLR* rs2569556, MAF = 0.190), the sample size needed was 184 [17]. We replicated the 45 variants in 677 participants (HLDL: 338, LLDL: 339). Similarly to the discovery phase, the participants were selected via nested case–control study design according to their TC and LDL-C levels. Genotyping was performed using an iPLEX^®^ Gold kit on the MassARRAY^®^ System (Agena Bioscience, San Diego, CA, USA) according to the manufacturer’s instructions. Data were analyzed using MassARRAY^®^ Typer v4.0 (Agena Bioscience). Two researchers manually inspected the genotyping reports for all samples independently. The genotypes of low-intensity variants were confirmed using Sanger sequencing using a BigDye™ Terminator v1.1 Cycle Sequencing Kit (Thermo Fisher Scientific) according to the manufacturer’s recommendations. The sequencing data were analyzed using Basic Local Alignment Search Tool (BLAST, http://blast.ncbi.nlm.nih.gov (accessed on 3 June 2020).

### 2.5. Statistical Analyses

Descriptive analysis for categorical data is reported as the frequency (*n)* and percentage (%). Continuous data are described as the mean and standard deviation (SD). Logistic regression modelling was initially performed to identify the association between hypercholesterolemia and individual genes or each environmental risk factor. To produce the most parsimonious model, variables showing no evidence of association (at *p* < 0.20) were removed, provided that the removal of the variable produced no substantive changes in the model. Predictive utility and gene–environment (i.e., genetic–non-genetic) interaction were assessed using the variables remaining in the final model. Using the final multivariate model, we estimated the increment in variance explained resulting from adding the genetic variants (risk-increasing or -decreasing) to the model that included non-genetic risk factors only. The risk explained by the risk factors was estimated using Nagelkerke’s pseudo R^2^. Based on the final model for each group, we calculated the area under the receiver operating characteristic (AUROC) curve and its 95% confidence interval (95% CI). These statistics measure logistic models’ predictive power and goodness-of-fit. They represent the accuracy with which a model can differentiate between two outcome categories, and thus measure the model’s potential diagnostic utility. An ideal test has an area under the curve (AUC) of 1, whereas random guessing would produce an AUC of 0.5. AUC ≥ 0.8 are often considered clinically useful. We assessed the multiplicative interaction between individual SNPs and non-genetic risk factors using logistic regression. All statistical analyses were performed with SPSS 20 (SPSS Inc., Chicago, IL, USA).

## 3. Results

### 3.1. Demographic Data and Non-Genetic Risk Factors Associated with Hypercholesterolemia

Table 1 shows the univariable analysis results of the clinical factors associated with hypercholesterolemia in Malays. The data were collected from the internal validation phase (*n* = 677). Age at baseline, fasting blood glucose, ever-use of tobacco products, diabetes mellitus (DM) with medication, and family history of hyperlipidemia were associated with increased hypercholesterolemia risk. The mean age at baseline of the HLDL participants was 53.36 (SD 6.36) years compared to the 51.63 (SD 6.46) years of the LLDL participants (*p* = 0.001). HLDL participants had higher fasting blood glucose [7.15 (SD 3.62) mg/L)] compared to LLDL participants [6.51 (SD 2.79) mg/L)] (*p* = 0.012). The use of tobacco products increased hypercholesterolemia risk by 1.66 times (*p* = 0.005). Interestingly, type 2 DM patients with metformin treatment had 38% reduced hypercholesterolemia risk (*p* = 0.015). Participants with a family history of hyperlipidemia had 2.44 times increased hypercholesterolemia risk (*p* = 0.028) compared to participants without a family history of hypercholesterolemia. There were no differences in body mass index (BMI), sex distribution, history of stroke, heart failure, obesity with medication, hypertension with medication, family history of hypertension, hyperlipidemia, heart disease, DM, and CVD between the HLDL and LLDL groups.

### 3.2. WES Identification of Risk-Increasing and -Reducing Variants

**Figure 1:**
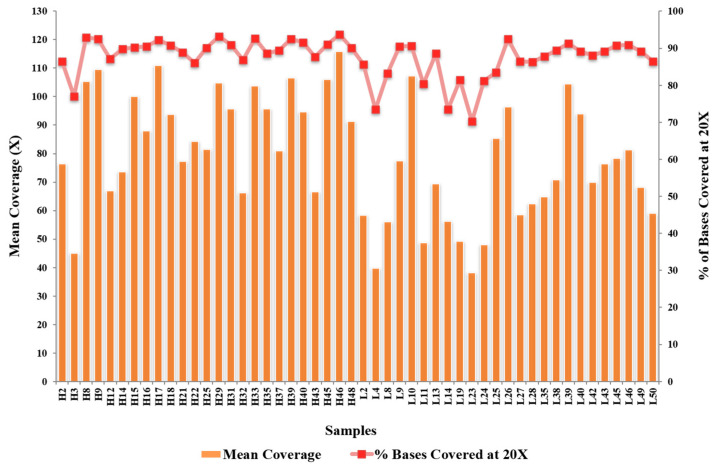
The statistics for the coverage of each sample.

The mean depth of WES for the 50 samples sequenced was 79.19×, with 87.64% of the exome covered at ≥20×. Figure 1 shows the statistics for the coverage of each sample. All samples passed the minimum 30× mean coverage, and 70% of the target regions were covered at 20× after we had removed duplicates. We identified five novel variations among the tier 1 genes: four frameshift deletions (*LDLR*: 1, *PCSK9*: 1, *LDLRAP1*: 2) and one non-frameshift substitution in *PCSK9*. In addition, 11 known variants were identified (non-synonymous mutations: four in *APOB* (rs376602710, rs1333175181, rs746414462, rs533617), six in *LDLR* (rs760436036, rs879254597, rs773658037, rs879254424, rs144172724, rs368708058), one in *PCSK9* (rs794728683)) (Table 2). These variants were identified only in the HLDL participants, suggesting that hypercholesterolemia in these patients could be due to genetic factors. Impact prediction of non-synonymous single-nucleotide variants at protein level revealed that seven mutations were damaging (*APOB*: p.A467G, p.T1222I, p.R1599H, p.H1923R; *PCSK9*: p.R215H; *LDLR*: p.E58G and p.E101K) and four were possibly damaging or tolerated. Based on the ClinVar database, five variants were pathogenic/likely pathogenic, two variants had conflicting pathogenicity, one variant was of uncertain significance, and one variant was benign/likely benign. Table 2 lists the variants identified in the tier 1 genes. Only 13 of the 25 HLDL participants carried tier 1 variants, and the number of samples per variant was relatively small.

We identified 76 risk-increasing variants in 25 tier 2 genes (Supplementary Table S5). In total, there were 65 novel variants and 11 known variants. The most common variants were in the *NYNRIN*, *CELSR2*, *PARP10*, *MAF1*, and *OSBPL7* (oxysterol-binding protein-like 7) genes (Table 3). These tier 2 genes are not directly implicated in FH, but are associated with LDL regulation and can affect the expression of the LDL-regulating genes. Hypercholesterolemia in these patients could be due to polygenic traits with non-genetic risk factor influences. We analyzed the risk-increasing variants among the tier 3 genes. There were 56 high-frequency variants in the HLDL group (>10 individuals/variant, ≥40%; these variants were not observed in the LLDL group) (Supplementary Table S6). Interestingly, 14 variants (novel: 11, known: 3) had very high-frequency samples per variant (15–19 or 60–76%) (Table 4). Similarly to the tier 2 gene variants, hypercholesterolemia in the patients with tier 3 gene variants could be due to polygenic traits with non-genetic risk factor influences.

**Figure 2:**
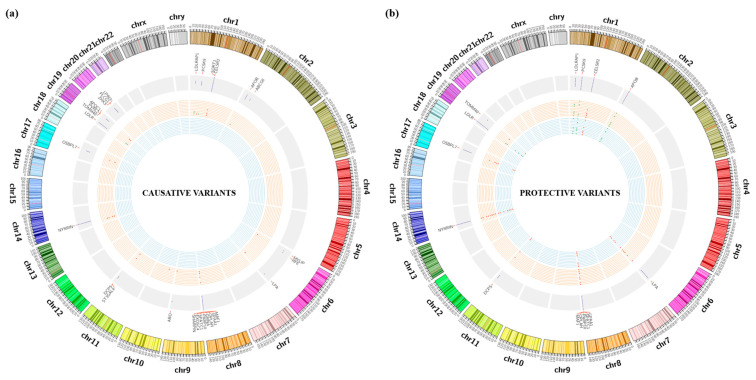
(**a**,**b**) Circus plot of the risk-increasing and -reducing variants identified through WES.

We also identified 108 risk-reducing variants from 40 genes. There were 17 variants (eight novel and nine known tier 1 protective variants: *APOB*: 9, *LDLR*: 1, *LDLRAP1*: 1, *PCSK9*: 5) (Supplementary Table S7). Sixty-two tier 2 variants (novel: 19, known: 43) were identified in 10 genes: *CELSR2*, *DCPS*, *GPAA1*, *LPA*, *MAF1*, *NYNRIN*, *OPLAH*, *OSBPL7*, *PARP10*, and *SPATC1* (Supplementary Table S8). In tier 3 genes, 23 variants (novel: 17, known: 6) were identified in 23 genes (Supplementary Table S9). Figure 2a,b illustrate the circus plot of the risk-increasing and -reducing variants identified through WES.

### 3.3. Variants and Association with Hypercholesterolemia Risk in Malaysian Malays

As both the HLDL and LLDL groups had relatively low prevalence of variants, we performed an internal validation study involving 677 participants. Table 5 shows the significant variants associated with HLDL in Malays. The *OSBPL7* variant c.651_652del: p.217_218del was associated with 16.89 times higher odds for hypercholesterolemia (*p* < 0.001). The *PCSK9* rs151193009 (c.C277T: p.R93C) variant was associated with low odds for hypercholesterolemia (*p* = 0.001).

### 3.4. Hypercholesterolemia Predictive Models Combining Genetic and Non-Genetic Risk Factors

In all three final models, age, fasting blood glucose, and type 2 diabetes on medication were associated with HLDL (Table 6). Participants who were 5 years older had 1.28–1.34 higher odds (odds ratio (OR): 1.055–1.065) for HLDL compared to participants who were 5 years younger. Participants with fasting blood sugar levels higher by 5 mmol/L had approximately two times higher odds (OR: 1.16–1.18) for HLDL. All three models also estimated that diabetic participants on medication had low odds for HLDL. Model 1, which consisted of the non-genetic factors, only explained 11% of the variation in the outcome of HLDL (Nagelkerke’s R2 = 0.11), with an AUC of 0.68 (95% CI: 0.64, 0.72).

In Model 2, participants with a family history of hyperlipidemia had three times higher odds for HLDL (OR: 3.30; 95% CI: 1.44, 7.56). Participants with the novel variant *OSBPL7*: c.651_652del: p.217_218del had almost 17 times higher odds for high HLDL compared to participants with the wild-type genotype. Incorporating this variant in Model 2 increased the ability of the model to explain the variation of having HLDL to 20.3% [AUC = 0.73 (95% CI: 0.69, 0.77)].

In Model 3, participants with the CT genotype in *PCSK9* rs151193009 had low odds (OR: 0.12; 95% CI: 0.03, 0.42) for HLDL compared to participants with the C genotype. Combining this variant with the non-genetic risk factors slightly increased the chances of HLDL to 14.3% [AUC = 0.69 (95% CI: 0.65, 0.74)]. There was no evidence of gene–environment (i.e., genetic–non-genetic) interaction between individual SNPs and each non-genetic risk factor.

These risk factors for hypercholesterolemia differ slightly between males and females (Supplementary Table S10). In males, history of tobacco use significantly increased risk of hypercholesterolemia by 1.9 times (OR: 1.90; 95% CI: 1.06, 3.37) while those who had diabetes with medication hade 57% reduced risk of hypercholesterolemia (OR: 0.43; 95% CI: 0.24, 0.79), while in females, the factors that increased risk of hypercholesterolemia were age, fasting blood glucose, history of tobacco use, and hyperlipidemia with medication. Similarly in males, diabetes with medication also reduced the risk of hypercholesterolemia in females. For the genetic risk factors, T2FH_*OSBPL7*_01 increased risk of hypercholesterolemia, while rs151193009 reduced the risk in both males and females. In addition, T2FH_*SPATC1*_01 reduced the risk of hypercholesterolemia only in males (Supplementary Table S11).

## 4. Discussion

In the present study, we identified the genetic and non-genetic risk factors associated with hypercholesterolemia in Malaysian Malays. In total, four of the 18 environmental factors analyzed were associated with increased LDL: age, tobacco use, fasting blood glucose level, and family history of hyperlipidemia, which is consistent with studies on other populations [24]. We identified a novel *OSBPL7* (c.651_652del) variant that increased the risk for hypercholesterolemia by 17 times. Patients with type 2 DM on medication and those with the *PCSK9* rs151193009 variant showed reduced risk of hypercholesterolemia. The combination of age, tobacco use, fasting blood glucose level, and family history of hyperlipidemia with *OSBPL7* c.651_652del increased the hypercholesterolemia risk from 11% to 20.3%. Recent studies showed that Malaysian Malays have the highest prevalence of elevated triglycerides and LDL-C in Malaysia [25], and are the second-ranked ethnicity with a high risk of developing cardiovascular disease (CVD) [26]. Importantly, ethnic Malays are the major contributor to the statistics of familial hypercholesterolemia (FH) [27], suggesting a higher influence of genetics on hypercholesterolemia in Malays. By incorporating the variants specific to Malays, these findings could form the basis for early genetic screening of hypercholesterolemia in Malaysia to reduce the morbidity and mortality from related cardiovascular complications.

We also showed that age, tobacco use, fasting blood glucose level, and family history of hyperlipidemia increased hypercholesterolemia risk in Malays. Older age is often associated with elevated levels of circulating lipids including LDL-C [28]. One explanation is the change in the lipolysis in adipocytes, in which the reduction of catecholamines and hormone-sensitive lipase by aging causes the adipocytes to reduce their uptakes of the circulating lipids for the storage [29]. Another is the aging-related change in the lipid synthesis pathways (lipolysis, lipid metabolism and lipid transport), in which aging reduces the capacity of the skeletal muscles to oxidize and metabolize the circulating lipids for energy [30]. The hepatic lipid metabolism is also shifted due to aging, whereby lipid synthesis is increased, and fatty acid oxidation is decreased, thus accumulating the lipid particles in the organ [31]. These changes in the metabolic rates subsequently cause increased HDL, LDL, and TG levels [24]. Moreover, aging also increases reactive oxidative species and reduces cellular antioxidant capacity, which leads to increased oxidative stress [32]. This activates 3-hydroxy-3-methylglutaryl–coenzyme A (HMG-CoA) reductase, which increases cholesterol synthesis and LDL-C levels by downregulating LDLR synthesis [32]. However, LDL-C levels also decrease at the age of 50–59 years, possibly due to the low ACAT2 (acetyl-CoA acetyltransferase 2) activity that causes lower very-LDL-C (VLDL-C) secretion and LDL-C production [32,33]. In the present study, patients with high fasting blood glucose or diabetes were more likely to have hypercholesterolemia. The presence of insulin resistance contributes to the dysregulation of lipid metabolism [34]. Thus, the use of diabetes medication such as metformin will likely improve LDL-C levels [35]. Metformin intake reduces blood LDL-C levels by activating adenosine monophosphate (AMP)-activated protein kinase and can suppress fatty acid desaturase (FADS) action [35]. Another hypercholesterolemia risk factor is family history of hyperlipidemia, which increases the risk for developing FH [7]. In the present study, only one lifestyle factor, i.e., tobacco consumption, was associated with higher hypercholesterolemia risk. Tobacco smoking is a known CVD risk factor and is associated with higher serum cholesterol, TG, and LDL-C levels [36]. Nicotine stimulates the production of adrenaline and causes higher serum concentrations of free fatty acids, further inducing hepatic regulation and cholesterol, VLDL, and TG production [36]. All of our non-genetic risk factors were also present in the other populations with high prevalence of hypercholesterolemia. For the Singaporean multi-ethnic population with 52.2% prevalence of hypercholesterolemia, the risk factors are the low education ≤6 years, current smokers, and blue-collar jobs or unemployment with greater unawareness of hypercholesterolemia [37]. In this study [37], the ethnic Malays had the highest risk factors, including for the prevalence of diabetes and hypertension. In another study in Thailand, the prevalence of hypercholesterolemia was 66.5% [38,39]. The regression analysis confirmed that the risk factors included older age, history of alcohol consumption, and family history of dyslipidemia [38,39]. In another Malay ethnic-majority country, Indonesia, the prevalence of hypercholesterolemia is 49.5%, and reported risk factors include the inadequate level of physical activity and smoking [40]. From these findings, the Malays in our study had risk factors in concordance with previous publications, and additional fasting blood glucose observed in our study may be due to the additional measurement that was made in our study but was missing in the other publications.

We identified 12 novel risk-increasing variants in tier 1 genes (*APOB*, *LDLR*, *LDLRAP1*, *PCSK9*). As mutations in FH genes are usually ethnicity-specific, the variants might occur in only Malays, but this observation requires validation in other ethnic groups. Four known variants were also identified: rs376602710, rs533617, rs144172724, and rs368708058 [41]. rs376602710 is a missense mutation with uncertain significance in familial hypobetalipoproteinemia (FHBL) and in FH. rs533617 is a missense variant that has been observed in several conditions, including hypercholesterolemia autosomal dominant type B, FH, and FHBL. Interpretations of its pathogenicity are conflicting; therefore, its role in hypercholesterolemia is unknown. rs144172724 is pathogenic and has been identified in patients with FH in Finland, the Netherlands, and France [42]. rs368708058 has been identified in patients with FH in the UK and the Netherlands [41]. The pathogenicity of this mutation is uncertain.

In the present study, 48% of the participants could be classified as probable/possible FH based on the Simon Broome criteria or as monogenic FH. Surprisingly, 13 participants (52%) did not have mutations in the FH-related tier 1 genes, suggesting polygenic inheritance. Hypercholesterolemia in patients with tier 2 or 3 gene variants could be due to polygenic traits with non-genetic risk factor influences. We identified 76 risk-increasing variants in 25 tier 2 genes, and the most common variants were in the *NYNRIN*, *CELSR2*, *PARP10*, *MAF1*, and *OSBPL7* genes. The tier 3 genes had a high frequency of variants (*n* = 15–19). Most of the tier 3 variants were novel, and only three variants, i.e., rs11243045, rs71557212, and rs1670534, had been identified previously [43]. However, the association of these tier 3 variants with hypercholesterolemia remains unknown. Despite that, these tier 3 variants were only identified in the HLDL participants and not among the LLDL group; thus, we postulate that they could play an important role in lipid metabolism in Malays with hypercholesterolemia.

Internal validation of the selected 45 variants in 677 Malay participants with hypercholesterolemia showed that a novel variant in *OSBPL7* (c.651_652del) increases hypercholesterolemia risk by 17 times. Overexpression of the *OSBPL7* gene can affect serum LDL and TG levels and hepatic TG synthesis [44]. This is possible via SREBP1C (sterol regulatory element–binding protein 1C) [44], a major regulator of lipogenesis. This *OSBPL7* variant is the first to be associated with hypercholesterolemia, particularly in Malays. Furthermore, the combination of age, tobacco use, fasting blood glucose level, and family history of hyperlipidemia with the presence of *OSBPL7* c.651_652del increased the hypercholesterolemia risk by 8.3%. Our study indicates that the *OSBPL7* variant might have greater genetic effects on Malay patients. Further studies are needed to understand the role of *OSBPL7* in lipid metabolism. The limitation of this study was the validation cohort. We used the same cohort in TMC project because the number of hypercholesterolemia patients in our biobank was insufficient for validation. We are currently recruiting samples of FH patients from the Hospital Chancellor Tuanku Muhriz obesity clinic. We hope to be able to validate these findings in the clinical samples in our future study.

Several variants that can reduce the risk of developing hypercholesterolemia were also identified from the LLDL group. In total, 139 were novel, whereas 120 were known; the most common protective variants were identified in the *CELSR2*, *LPA*, *NYNRIN*, *OPLAH*, *PARP10*, *PCSK9*, and *SPATC1* genes. *PCSK9* rs151193009 reduced hypercholesterolemia risk in Malays, consistent with previous findings on the protectiveness of this variant against high LDL-C and coronary artery disease risk in Asians only [45,46]. *PCSK9* is a serine protease that regulates LDLR levels by degradation, and the rs151193009 variant causes *PCSK9* loss of function, which in return increases hepatic LDLR expression. Consequently, there is greater removal of cholesterol-rich LDL particles from the plasma [47].

## 5. Conclusions

We have identified the genetic variants associated with hypercholesterolemia risk in Malaysian Malays. Non-genetic risk factors such as age, fasting blood glucose level, history of use of tobacco products, and family history of hyperlipidemia are also associated with hypercholesterolemia. A panel of hypercholesterolemia-associated variants in Malays could be developed for early diagnosis of FH and family screening. Identifying the variants associated with hypercholesterolemia may aid individual risk stratification for hypercholesterolemia for early intervention and disease management.

## Figures and Tables

**Table 1:** Univariable analysis of the clinical factors associated with HLDL versus LLDL from the internal validation cohort.

Clinical Factor		High LDL	Low LDL	cOR (95% CI)	χ^2^ (df)	*p*-Value
		Mean (SD)	Mean (SD)			
Age at baseline		53.36 (6.36)	51.63 (6.45)	1.04 (1.02, 1.07)	11.94 (1)	0.001 *
Fasting blood glucose (mg/L)		7.15 (3.62)	6.51 (2.79)	1.07 (1.01, 1.12)	6.30 (1)	0.012 *
BMI (kg/m^2^)		26.71 (4.15)	26.65 (5.19)	1.00 (0.97, 1.04)	0.03 (1)	0.854
		***n*** (%)	***n*** (%)			
Sex	Male	134 (39.6)	113 (33.3)	1.31 (0.96, 1.80)	2.90 (1)	0.088
	Female (Ref.)	204 (60.4)	226 (66.7)	1		0.09
Tobacco product use	Yes	102 (30.9)	70 (21.2)	1.66 (1.17, 2.36)	7.98 (1)	0.005 *
	No (Ref.)	228 (69.1)	260 (78.8)	1		
Stroke diagnosis history	Yes	3 (0.9)	7 (2.1)	0.43 (0.11, 1.66)	1.51 (1)	0.219
	No (Ref.)	334 (99.1)	332 (97.9)	1		
Angina or myocardial infarction diagnosis history	Yes	11 (3.3)	11 (3.2)	1.00 (0.43, 2.35)	0	0.989
	No (Ref.)	326 (96.7)	328 (96.8)	1		
Heart failure diagnosis history	Yes	7 (2.1)	11 (3.2)	0.63 (0.24, 1.65)	0.88 (1)	0.35
	No (Ref.)	330 (97.9)	328 (96.8)	1		
Obesity with medication status	Yes	26 (7.7)	36 (10.6)	0.70 (0.42, 1.19)	1.70 (1)	0.193
	No (Ref.)	311 (92.3)	303 (89.4)	1		
Hypertension with medication status	Yes	113 (33.5)	127 (37.5)	0.84 (0.61, 1.15)	1.14 (1)	0.286
	No (Ref.)	224 (66.5)	212 (62.5)	1		
Diabetes mellitus with medication status	Yes	55 (16.3)	81 (23.9)	0.62 (0.42, 0.91)	5.97 (1)	0.015 *
	No (Ref.)	282 (83.17)	258 (76.1)	1		
Family history of hypertension	Yes	106 (31.5)	118 (34.8)	0.86 (0.62, 1.18)	0.86 (1)	0.354
	No (Ref.)	231 (68.5)	221 (65.2)	1		
Family history of diabetes mellitus	Yes	86 (25.5)	92 (27.1)	0.92 (0.65, 1.30)	0.23 (1)	0.633
	No (Ref.)	251 (74.5)	247 (72.9)	1		
Family history of stroke	Yes	29 (8.6)	40 (11.8)	0.70 (0.43, 1.17)	1.87 (1)	0.704
	No (Ref.)	308 (91.4)	299 (88.2)	1		
Family history of angina	Yes	18 (5.3)	10 (2.9)	1.86 (0.84, 4.08)	2.37 (1)	0.124
	No (Ref.)	319 (94.7)	329 (97.1)	1		
Family history of hyperlipidemia	Yes	21 (6.2)	9 (2.7)	2.44 (1.20, 5.40)	4.81 (1)	0.028 *
	No (Ref.)	316 (93.8)	330 (97.3)	1		
Family history of heart disease	Yes	42 (12.5)	31 (9.1)	1.42 (0.87, 2.31)	1.92 (1)	0.166
	No (Ref.)	295 (87.5)	308 (90.9)	1		
Family history of cardiovascular disease	Yes	76 (22.6)	71 (20.9)	1.20 (0.76, 1.58)	0.26 (1)	0.612
	No (Ref.)	261 (77.4)	268 (79.1)	1		

* *p*-value significant at <0·05. Ref, reference; LDL, low-density lipoprotein; BMI, body mass index; cOR, 95% CI, 95% confidence interval.

**Table 2:** The tier-1 variants in Malaysian Malays with hypercholesterolemia detected via WES.

Chromosome	Gene	Nomenclature	Variant Type	snp138	Clinical Significance (ClinVar)	Frequency in HLDL, *n*	Sample ID
2	*APOB*	NM_000384.3:c.C1400G;p.A467G	Non-synonymous SNV	rs376602710	Uncertain significance	1	H39
2	*APOB*	NM_000384.3:c.C3665T:p.T1222I	Non-synonymous SNV	rs1333175181	-	1	H17
2	*APOB*	NM_000384.3:c.G4796A:p.R1599H	Non-synonymous SNV	rs746414462	Conflicting pathogenicity	1	H33
2	*APOB*	NM_000384.3:c.A5768G:p.H1923R	Non-synonymous SNV	rs533617	Benign/Likely benign	1	H48
19	*LDLR*	NM_000527.5:c.1994_2010del:p.665_670del	Frameshift deletion	-	-	1	H14; H40
19	*LDLR*	NM_000527.5:c.T2037A;p.Y679X	Stop-gain SNV	rs760436036	Pathogenic	1	H15; H16
19	*LDLR*	NM_000527.5:c.G622A;p.E208K	Non-synonymous SNV	rs879254597	Pathogenic/Likely pathogenic	2	H9
19	*LDLR*	NM_000527.5:c.G1247T:p.R416L	Non-synonymous SNV	rs773658037	Likely pathogenic	1	H39
19	*LDLR*	NM_000527.5:c.A173G:p.E58G	Non-synonymous SNV	rs879254424	Likely pathogenic	2	H45
19	*LDLR*	NM_000527.5:c.G301A:p.E101K	Non-synonymous SNV	rs144172724	Pathogenic/Likely pathogenic	1	H33
19	*LDLR*	NM_000527.5:c.C1284G:p.N428K	Non-synonymous SNV	rs368708058	Likely benign	1	H32
1	*LDLRAP1*	NM_015627.3:c.603_604del:p.201_202del	Frameshift deletion	-	-	2	H18; H48
1	*LDLRAP1*	NM_015627.3:c.584delC:p.A195fs	Frameshift deletion	-	-	2	H18
1	*PCSK9*	NM_174936.4:c79delG:p.G27fs	Frameshift deletion	-	-	1	H33
1	*PCSK9*	NM_174936.4:c.G644A:p.R215H	Non-synonymous SNV	rs794728683	Conflicting pathogenicity	1	H32
1	*PCSK9*	NM_174936.4:c.77_79CGC	Non-frameshift substitution	-	-	1	H33

HLDL, high LDL-C group; SNV, single-nucleotide variant.

**Table 3:** The most common tier 2 variants.

Gene	Variants, *n*	Frequency in HLDL, *n*	Function
NYNRIN	12	13	NYN domain and retroviral integrase containing protein.
CELSR2	11	12	A member of the flamingo subfamily, part of the cadherin superfamily. It is postulated that these proteins are receptors involved in contact-mediated communication.
PARP10	6	9	Poly (ADP-ribose) polymerases (PARPs), such as PARP10, regulate gene transcription by altering chromatin organization by adding ADP-ribose to histones. PARPs can also function as transcriptional cofactors.
MAF1	1	7	Repressor of RNA polymerase III transcription MAF1 homolog.
OSBPL7	3	6	A member of the oxysterol-binding protein (OSBP) family, a group of intracellular lipid receptors.

HLDL, high LDL-C group.

**Table 4:** The most common tier 3 variants identified.

Chromosome	Gene	Nomenclature	Variant Type	snp138	Clinical Significance (ClinVar)	Frequency in HLDL, *n*	Frequency in LLDL, *n*
11	*WDR74*	NM_018093.3:c.855delC:p.A285fs	Frameshift deletion	-	-	19	0
20	*FRG1BP*	NR_145491.1:n.509TTG	Nonframeshift insertion	rs112430454	-	18	0
3	*SEC13*	NM_183352.3:c.950delG:p.G317fs	Frameshift deletion	-	-	18	0
19	*KEAP1*	NM_203500.2:c.966delC:p.P322fs	Frameshift deletion	-	-	18	0
1	*C1orf85/* *GLMP*	NM_144580.3:c.35_36del:p.12_12del	Frameshift deletion	-	-	17	0
7	*MUC12*	NM_001164462.2:c.A5251C:p.T1751P	Nonsynonymous SNV	rs71557212	-	17	0
15	*UBR1*	NM_174916.3:c.4519delC:p.P1507fs	Frameshift deletion	-	-	17	0
1	*GBP7*	NM_207398.3:c.460delG:p.A154fs	Frameshift deletion	-	-	16	0
4	*RNF212*	NM_001193318.3:c.A784G:p.I262V	Nonsynonymous SNV	rs1670534	-	16	0
6	*DPCR1/MUCL3*	NM_080870.4:c.2453delC:p.S818fs	Frameshift deletion	-	-	16	0
14	*HEATR5A*	NM_015473.4:c.2656delG:p.V886fs	Frameshift deletion	-	-	15	0
14	*HEATR5A*	NM_015473.4:c.2656_2657AT	Non-frameshift substitution	-	-	15	0
12	*TIMELESS*	NM_003920.5:c.3280delG:p.A1094fs	Frameshift deletion	-	-	15	0
16	*GTF3C1*	NM_001520.4:c.167delG:p.G56fs	Frameshift deletion	-	-	15	0

HLDL, high LDL-C group; LLDL, low LDL-C group.

**Table 5:** Univariable analysis of SNPs associated with HLDL versus LLDL.

Gene	Changes/Type	rs#	Genotype	cOR (95% CI)	χ^2^ (df)	*p*-Value
*APOB*	NM_000384.3:c.A5768G:p.H1923R	rs533617	T (Ref)	1		
	Nonsynonymous SNV		TC	2.37 (0.61, 9.24)	1.54 (1)	0.214
*APOB*	NM_000384.3:c.G4796A:p.R1599H	rs746414462	C (Ref)	1		
	Nonsynonymous SNV		CT	3.04 (0.06, 15.15)	3 (1)	0.176
*APOB*	NM_000384.3:c.C1400G:p.A467G	rs376602710	G (Ref)	1		
	Nonsynonymous SNV		GC	0.14 (0.02, 1.15)	3.36 (1)	0.067
*LDLRAP1*	NM_015627.3:c.603_604del:p.201-203del Frameshift deletion	Novel	T (Ref)	1		
			CT	0.90 (0.65, 1,25)	0.41 (1)	0.521
			C	1.11 (0.70, 1.77)	0.21 (1)	0.644
*CELSR2*	NM_001408.3:c.C6517T:p.R2173C	rs142780237	C (Ref)	1		
	Nonsynonymous SNV		CT	3.20 (0.64, 15.98)	2.01 (1)	0.156
*CELSR2*	NM_001408.3:c.6756delC:p.Y2252fs	Novel	C (Ref)	1		
	Frameshift deletion		G	1.15 (0.07, 18.49)	0.01 (1)	0.921
*LPA*	NM_005577.4:c.A4195C:p.T1399P	rs41272100	T (Ref)	1		
	Nonsynonymous SNV		TG	0.58 (0.17, 2.00)	0.74 (1)	0.389
*MAF1*	NM_032272.5:c.532delG: p.G178fs	Novel	G (Ref)	1		
	Frameshift deletion		GA	0.57 (0.26, 1.26)	1.94 (1)	0.164
*NYNRIN*	NM_025081.3:c.169_173GC	Novel	G (Ref)	1		
	Non-frameshift deletion		GC	1.15 (0.07, 18.54)	0.01 (1)	0.919
*OSBPL7*	NM_145798.3:c.651_652del:p217_218del	Novel	C (Ref)	1		
	Frameshift deletion		CA	16.89 (6.05, 47.12)	29.15 (1)	<0.001 *
*OSBPL7*	NM_145798.3:c.348_352ACCCT Non-frameshift substitution	Novel	C (Ref)	1		
			CT	0.63 (0.36, 1.09)	2.72 (1)	0.099
			T	0.96 (0.06, 15.39)	0.01 (1)	0.976
*SPATC1*	NM_198572.3:c.C1504A:p.Q502K	rs210925917	C (Ref)	1		
	Nonsynonymous SNV		CA	0.43 (0.17, 1.05)	3.45 (1)	0.063
*PARP10*	NM_032789.5:c.283delG	Novel	G (Ref)	1		
	Frameshift deletion		GDEL	1.61 (0.73, 3.58)	1.39 (1)	0.238
*APOB*	NM_000384.3:c.G4163A:p.R1388H Nonsynonymous SNV	rs13306187	C (Ref)	1		
			CT	1.40 (0.68, 2.89)	0.81 (1)	0.367
			T			
*PCSK9*	NM_174936.4:c.C277T:p.R93C Nonsynonymous SNV	rs151193009	C (Ref)			
			CT	0.12 (0.04, 0.41)	11.72 (1)	0.001 *
			T			
*APOB*	NM_000384.3:c.G7331A:p.R2444H	rs200143030	C (Ref)	1.14 (0.23, 5.67)	0.02 (1)	0.877
	Nonsynonymous SNV		CT			
*LDLR*	c.C4T:p.R2X Stop-gain SNV	rs2228671	C (Ref)	1		
			CT	0.67 (0.34, 1.33)	1.32 (1)	0.251
			T			
*LPA*	NM_005577.4:c.C6046T:p.R2016C Nonsynonymous SNV	rs3124784	G (Ref)	1		
			GA	1.34 (0.90, 1.99)	2.13 (1)	0.145
			A	0.40 (0.04, 3.87)	0.63 (1)	0.429
*LPA*	NM_005577.4:c.A5673G:p.I1891M Nonsynonymous SNV	rs3798220	T (Ref)	1		
			TC	0.56 (0.34, 1.05)	3.23 (1)	0.072
			C			
*LDLR*	c.G224A:p.G75D Nonsynonymous SNV	rs3826810	G (Ref)	1		
			GA	0.65 (0.42, 0.99)	4.09 (1)	0.043
			A			
*SPATC1*	NM_198572.3:c.T193C:p.S65P	rs60050811	T (Ref)	1		
	Nonsynonymous SNV		TC	0.89 (0.40, 1.99)	0.09 (1)	0.770
*NYNRIN*	NM_025081.3c.A823G:p.S275G Nonsynonymous SNV	rs74036628	A (Ref)	1		
			AG	0.89 (0.62, 1.27)	0.44 (1)	0.505
			G	0.68 (0.28, 1.68)	0.69 (1)	0.406

* *p*-value significant at <0.002 (0.05/27) after Bonferroni correction. Ref, reference; cOR, 95% CI, 95% confidence interval; df, degrees of freedom.

**Table 6:** Multiple logistic regression models of factors associated with HLDL.

Factor	Model 1		Model 2		Model 3	
	aOR (95% CI)	*p*-Value	aOR (95% CI)	*p*-Value	aOR (95% CI)	*p*-Value
Age (years)	1.04 (1.02, 1.07)	0.001 *	1.04 (1.01, 1.07)	0.004 *	1.05 (1.02, 1.08)	0.001 *
Fasting blood glucose (mmol/L)	1.16 (1.09, 1.24)	<0.001 *	1.17 (1.09, 1.25)	<0.001 *	1.15 (1.07, 1.23)	<0.001 *
Ever-use of tobacco products (yes)	1.60 (1.08, 2.38)	0.019	1.52 (1.01, 2.28)	0.045	1.69 (1.12, 2.55)	0.013
Diabetes with medication status (yes)	0.27 (0.16, 0.45)	<0.001 *	0.26 (0.15, 0.44)	<0.001 *	0.29 (0.17, 0.50)	<0.001 *
Family history of hyperlipidemia (yes)	2.94 (1.30, 6.68)	0.010	3.30 (1.44, 7.56)	0.010	2.19 (0.90, 5.35)	0.084
T2FH_*OSBPL7*_01 (novel) (CA vs. C)			16.60 (5.82, 47.35)	<0.001 *		
rs151193009 (CT vs. C)					0.12 (0.03, 0.42)	0.001 *
Nagelkerke’s R2	0.11		0.20		0.14	
AUC (95% CI)	0.68 (0.64, 0.72)		0.73 (0.69, 0.77)		0.69 (0.65, 0.74)	

Model 1: Clinical risk factors only; Model 2: clinical risk factors + increased risk variant (*OSBPL7*_01); Model 3: clinical risk factors + reduced risk variant (rs15113009). aOR, adjusted odds ratio; 95% CI, 95% confidence interval; AUC, area under the curve, *p*-value < 0.05 is significant.

## Data Availability

The WES data generated in this study have been submitted to the NCBI SRA under accession number PRJNA607111 (https://www.ncbi.nlm.nih.gov/bioproject/?term=PRJNA607111 (accessed on 17 Feburary 2020).

## Supplementary Materials

The following supporting information can be downloaded at: https://www.mdpi.com/article/10.3390/genes14030721/s1, Table S1. Summary of the 25 hypercholesterolemic (HLDL) individuals for whole-exome sequencing. Based on the Simon Broome’s criteria, the cut-off point for hypercholesterolemia are the low-density lipoprotein C (LDL-C) level (>4.9 mmol/L and total cholesterol (TC) level (>7.5 mmol/L); Table S2. Summary of the 25 normal (LLDL) individuals for whole-exome sequencing. Based on the Simon Broome’s criteria, the cut-off point for hypercholesterolemia are the low-density lipoprotein C (LDL-C) level (<5.2 mmol/L and total cholesterol (TC) level (2.6–3.4 mmol/L); Table S3. Summary of the 27 causative variants that were chosen for validation in 677 individuals; Table S4. Summary of the 18 protective variants that were chosen for validation in 677 individuals; Table S5. Summary of the 76 causative variants in 25 of tier-2 genes identified from whole-exome sequencing; Table S6. Summary of the 56 causative variants in 54 of tier-3 genes identified from whole-exome sequencing; Table S7. Summary of the 17 protective variants in tier-1 genes identified from whole-exome sequencing; Table S8. Summary of the 62 protective variants in 10 tier-2 genes identified from whole-exome sequencing; Table S9. Summary of the 23 protective variants in 23 tier-3 genes identified from whole-exome sequencing; Table S10. Multiple logistic regression models of non-genetic risk factors associated with HLDL in males and females; Table S11. Multiple logistic regression models of genetic risk factors associated with HLDL in males and females.
