# Supplementary material for: Hypercholesterolemia in the Malaysian Cohort Participants: Genetic and Non-Genetic Risk Factors
Source: Genes (Basel). 2023 Mar 15;14(3):721. doi: 10.3390/genes14030721 (PMC10048611; doi:10.3390/genes14030721)
Supplement: Supplementary file 1 [file genes-14-00721-s001.zip › genes-2217247-supplementary.pdf]

Supplementary tables

Supplementary Table S1. Summary of the 25 hypercholesterolemic (HLDL) individuals for whole-exome sequencing.

Based on the Simon Broome's criteria, the cut-off point for hypercholesterolemia are the low-density lipoprotein C (LDL-C) level (>4.9 mmol/L and total cholesterol (TC) level (>7.5 mmol/L).

Sample Name	Sample ID	Age (years)	Gender	TC (mmol/L)	LDL-C (mmol/L)
H12	1811080107	50	Male	10.92	7.70
H3	511080024	62	Female	10.60	7.79
H22	1001111011	48	Male	11.12	8.62
H39	605123137	59	Female	10.65	8.74
H40	1305122033	36	Female	11.22	8.76
H43	1006122103	49	Female	11.33	8.84
H37	104122038	46	Female	10.96	8.88
H46	3007123035	50	Male	10.67	9.00
H2	211083117	46	Male	11.14	9.15
H35	912103030	49	Male	11.30	9.20
H45	2806122134	43	Male	11.18	9.22
H32	1209112009	59	Female	11.92	9.37
H8	1006080015	54	Male	11.25	9.61
H21	1101111003	51	Male	17.04	9.64
H48	2509122027	61	Male	11.97	9.96
H29	1107110011	60	Female	13.07	10.01
H16	1507100009	62	Female	11.86	10.13
H9	1706081005	62	Male	12.65	10.64
H25	2706110012	50	Female	13.01	10.75
H33	2810103001	51	Male	12.63	10.90
H15	1507100010	43	Female	12.73	11.00
H14	1307100008	57	Female	13.78	11.76
H31	2601115108	48	Female	14.19	12.07
H18	1110103020	58	Female	16.80	14.68
H17	2907100031	50	Female	18.01	16.09

Supplementary Table S2. Summary of the 25 normal (LLDL) individuals for whole-exome sequencing. Based on the Simon Broome's criteria, the cut-off point for hypercholesterolemia are the low-density lipoprotein C (LDL-C) level (<5.2 mmol/L and total cholesterol (TC) level (2.6–3.4 mmol/L).

Sample	Sample ID (TMC)	Age (years)	Gender	TC (mmol/L)	LDL-C (mmol/L)
L14	808105116	38	Male	2.66	0.45
L49	1508123006	41	Male	2.43	0.64
L27	2305111037	43	Male	2.54	0.80
L26	3101113010	62	Male	3.01	0.81
L38	1204120025	59	Male	2.25	0.82
L19	703111020	39	Male	2.42	0.86
L11	1507101017	49	Female	2.45	0.87
L2	1606080132	55	Female	2.98	0.92
L24	405111026	54	Male	2.68	0.98
L50	3003111037	36	Female	2.96	1.05
L46	2605123110	54	Male	2.78	1.15
L28	3005116127	43	Male	2.49	1.20
L45	2005122013	53	Male	2.92	1.20
L23	2103115106	47	Female	2.84	1.23
L13	2807100022	57	Male	2.83	1.25
L42	1604120013	49	Female	3.01	1.26
L43	1604121009	59	Male	3.04	1.26
L9	2406103122	61	Male	3.01	1.30
L39	1704120005	53	Male	2.55	1.37
L4	412080030	48	Female	2.98	1.38
L25	1105116134	51	Male	2.89	1.44
L40	1904123116	47	Female	2.85	1.55
L8	1208071049	53	Female	2.91	1.73
L35	3101121004	60	Male	2.98	1.83
L10	1507100008	52	Female	2.58	1.85

Supplementary Table S3. Summary of the 27 causative variants that were chosen for validation in 677 individuals.

Gene	Exon	Base change	Amino acid change	SIFT prediction	Polyphen2 prediction	snp138	Clinical Significance (ClinVar)	Frequency in HLDL, n	Frequency in LLDL, n
APOB	26	NM_000384.3:c.A5768G	H1923R	Deleterious	Damaging	rs533617	Benign/Likely benign	1	0
APOB	26	NM_000384.3:c.G4796A	R1599H	Deleterious	Damaging	rs746414462	Conflicting pathogenicity	1	0
APOB	23	NM_000384.3:c.C3665T	T12221	Deleterious	Damaging	rs1333175181	-	1	0
APOB	11	NM_000384.3:c.C1400G	A467G	Deleterious	Damaging	rs376602710	Uncertain significance	1	0
LDLR	2	NM_000527.5:c.A173G	E58G	Deleterious	Damaging	rs879254424	Likely pathogenic	1	0
LDLR	3	NM_000527.5:c.G301A	E101K	Deleterious	Damaging	rs144172724	Pathogenic/Likely pathogenic	1	0
LDLR	3	NM_000527.5:c.G622A	E208K	Tolerated	Damaging	rs879254597	Pathogenic/Likely pathogenic	1	0
LDLR	7	NM_000527.5:c.C1284G	N428K	Deleterious	Possible Damaging	rs368708058	Uncertain significance	1	0
LDLR	12	NM_000527.5:c.T2037A	Y679X	-	-	rs760436036	Pathogenic	2	0
LDLRAP1	1	NM_015627.3:c.603_604del	201_202del	-	-	-	-	2	0
PCSK9	1	NM_174936.4:c.77_79CGC	-	-	-	-	-	1	0
PCSK9	4	NM_174936.4:c.G644A	R215H	Deleterious	Damaging	rs794728683	Conflicting pathogenicity	1	0
CELSR2	19	NM_001408.3:c.C6517T	R2173C	Tolerated	Damaging	rs142780237	-	2	0
CELSR2	20	NM_001408.3:c.6756delC	Y2252fs	-	-	-	-	2	0
CELSR2	25	NM_001408.3:c.7583delC	A2528fs	-	-	-	-	4	0
DCPS	5	NM_014026.6:c.666delC	C222fs	-	-	-	-	4	0
GPAA1	5	NM_003801.4:c.702delC	S234fs	-	-	-	-	2	0
LPA	27	NM_005577.4:c.A4195C	T1399P	Deleterious	Damaging	rs41272110	-	2	0
MAF1	6	NM_032272.5:c.532delG	G178fs	-	-	-	-	7	0
NYNRIN	2	NM_025081.3:c.171_173del	57_58del	-	-	-	-	1	0
OPLAH	27	NM_017570.5:c.3668delA	N1223fs	-	-	-	-	2	0
OPLAH	16	NM_017570.5:c.2189_2195GCCCCA	-	-	-	-	-	4	0

OSBPL7	7	NM_145798.3:c.651_652del	217_218del	-	-	-	-	5	0
OSBPL7	4	NM_145798.3:c.348_352ACCCT	-	-	-	-	-	2	0
PARP10	1	NM_032789.5:c.146delG	G49fs	-	-	-	-	4	0
SPATC1	5	NM_198572.3:c.C1504A	Q502K	Deleterious	Damaging	rs201925917	-	2	0
TOMM40	1	NM_001128917.2:c.240delG	P80fs	-	-	-	-	2	0

Abbreviations: LDL-C; low density lipoprotein, HLDL; high LDL-C group, LLDL; low LDL-C group.

Supplementary Table S4. Summary of the 18 protective variants that were chosen for validation in 677 individuals.

Gene	Exon	Base change	Amino acid change	SIFT prediction	Polyphen2 prediction	snp138	Clinical Significance (ClinVar)	Frequency in HLDL, n	Frequency in LLDL, n
APOB	25	NM_000384.3:c.G4163A	R1388H	Tolerated	Benign	rs13306187	Conflicting pathogenicity	0	2
APOB	26	NM_000384.3:c.G7331A	R2444H	Tolerated	Benign	rs200143030	Conflicting pathogenicity	0	2
LDLR	2	NM_000527.5:c.81C>T	C27=	-	-	rs2228671	Benign	1	3
LDLR	3	NM_000527.5:c.*141=	3 Prime UTR	-	-	rs875989890	Benign	1	5
LDLRAP1	1	NM_015627.3:c.T604C	S202P	Tolerated	Benign	rs6687605	Benign	5	9
PCSK9	1	NM_174936.4:c.83delC	A28fs	-	-	-	-	0	2
PCSK9	2	NM_174936.4:c.C277T	R93C	Tolerated	Damaging	rs151193009	Conflicting pathogenicity	0	3
CELSR2	1	NM_001408.3:c.1505delC	A502fs	-	-	-	-	0	2
CELSR2	8	NM_001408.3:c.G4799C	C1600S	Deleterious	Damaging	-	-	2	4
LPA	21	NM_005577.4:c.3148delG	G1050fs	-	-	-	-	0	4
LPA	37	NM_005577.4:c.A5673G	I1891M	Deleterious	Damaging	rs3798220	Benign	0	3
LPA	40	NM_005577.4:c.C6046T	R2016C	Tolerated	Damaging	rs3124784	-	4	8
MAF1	7	NM_032272.5:c.G707A	G236E	Tolerated	Benign	-	-	4	7
NYNRIN	2	NM_025081.3:c.168_169del	56_57del	-	-	-	-	0	2
NYNRIN	3	NM_025081.3:c.A823G	S275G	Tolerated	Benign	rs74036628	-	4	7
NYNRIN	4	NM_025081.3:c.2249delG	G750fs	-	-	-	-	0	2
PARP10	3	NM_032789.5:c.283delG	D95fs	-	-	-	-	0	2
SPATC1	1	NM_198572.3:c.T193C	S56P	Tolerated	Benign	rs60050811		0	2

* **Abbreviations:** LDL-C; low density lipoprotein, HLDL; high LDL-C group, LLDL; low LDL-C group.

Supplementary Table S5. Summary of the 76 causative variants in 25 of tier-2 genes identified from whole-exome sequencing.

Gene	Variant Type	Nucleotide and amino acid changes	SIFT prediction	Polyphen2 prediction	snp138	Clinical Significance (ClinVar)	Sample IDs	Frequency in HLDL, n	Frequency in LLDL, n
ABCG8	frameshift deletion	NM_022437.3:c.690delC:p.N230fs	-	-	-		H32	1	0
ABCG8	nonsynonymous SNV	NM_022437.3:c.C154T:p.L52F	Deleterious	Damaging	rs142250628	Benign	H40	1	0
ABO	nonsynonymous SNV	NM_020469.3:c.A259G:p.T87A	-	-	rs77641731	-	H8	1	0
APOC1	frameshift deletion	NM_001645.5:c.71delC:p.A24fs,	-	-	-	-	H22	1	0
CELSR2	frameshift deletion	NM_001408.3:c.7583delC:p.A2528fs	-	-	-	-	H17;H29;H31;H35	4	0
CELSR2	frameshift deletion	NM_001408.3:c.6756delC:p.Y2252fs	-	-	-	-	H25;H9	2	0
CELSR2	nonsynonymous SNV	NM_001408.3:c.C6517T:p.R2173C	Tolerated	Damaging	rs142780237	-	H15;H16	2	0
CELSR2	frameshift deletion	NM_001408.3:c.2991delG:p.Q997fs	-	-	-	-	H48	1	0
CELSR2	frameshift deletion	NM_001408.3:c.1412delC:p.T471fs	-	-	-	-	H25	1	0
CELSR2	frameshift deletion	NM_001408.3:c.8721delG:p.V2907fs	-	-	-	-	H8	1	0
CELSR2	frameshift deletion	NM_001408.3:c.8463delG:p.E2821fs	-	-	-	-	H8	1	0
CELSR2	frameshift deletion	NM_001408.3:c.6341delC:p.A2114fs	-	-	-	-	H43	1	0
CELSR2	frameshift deletion	NM_001408.3:c.7631delC:p.A2544fs	-	-	-	-	H29	1	0
CELSR2	frameshift deletion	NM_001408.3:c.6583delC:p.P2195fs	-	-	-	-	H14	1	0
CELSR2	nonframeshift substitution	NM_001408.3:c.7630_7631GG	-	-	-	-	H29	1	0
DCPS	frameshift deletion	NM_014026.6:c.666delC:p.C222fs	-	-	-	-	H14;H18;H39;H40	4	0
EXOSC4	frameshift deletion	NM_019037.3:c.506delC:p.A169fs	-	-	-	-	H32	1	0
GPAA1	frameshift deletion	NM_003801.4:c.702delC:p.S234fs	-	-	-	-	H21;H8	2	0
GPAA1	frameshift deletion	NM_003801.4:c.639delG:p.Q213fs	-	-	-	-	H31	1	0
GPAA1	nonsynonymous SNV	NM_003801.4:c.G1387A:p.A463T	Tolerated	Damaging	-	-	H37	1	0
GRINA	frameshift insertion	NM_000837.2:c.72_73insGC:p.G24fs	-	-	-	-	H46	1	0
GRINA	nonsynonymous SNV	NM_000837.2:c.C54A:p.N18K	Deleterious	Possible Damaging	-	-	H43	1	0
GRINA	frameshift deletion	NM_000837.2:c.275delG:p.G92fs	-	-	-	-	H14	1	0
GRINA	nonframeshift substitution	NM_000837.2:c.272_275AGGC	-	-	-	-	H14	1	0
GRINA	nonframeshift substitution	NM_000837.2:c.87_88TG	-	-	-	-	H39	1	0
HFE	nonsynonymous SNV	NM_000410.4:c.G845A:p.C282Y	Deleterious	Damaging	rs1800562	Conflicting pathogenicity	H2	1	0
LPA	nonsynonymous SNV	NM_005577.4:c.A4195C:p.T1399P	Deleterious	Damaging	rs41272110	-	H14;H9	2	0

LPIN3	frameshift deletion	NM_001301860.2:c.1197delC:p.F399fs	-	-	-	-	H40	1	0
LPIN3	frameshift deletion	NM_001301860.2:exon16:c.2010delG:p.Q670fs	-	-	-	-	H31	1	0
LPIN3	nonsynonymous SNV	NM_001301860.2:c.C881T:p.P294L	-	Possible Damaging	-	-		1	0
MAF1	frameshift deletion	NM_032272.5:c.532delG:p.G178fs	-	-	-	-	H18;H21;H25;H31;H35;H37;H8	7	0
MYLIP	nonsynonymous SNV	NM_013262.4:c.C1210T:p.H404Y	Deleterious	Damaging	-	-	H17	1	0
NYNRIN	nonframeshift substitution	NM_025081.3:c.169_173GC	-	-	-	-	H29;H31;H32;H33;H43	5	0
NYNRIN	frameshift substitution	NM_025081.3:c.169_173GGC	-	-	-	-	H29;H31;H32;H33	4	0
NYNRIN	nonframeshift substitution	NM_025081.3:c.167_172AGC	-	-	-	-	H16;H22;H37;H40	4	0
NYNRIN	frameshift deletion	NM_025081.3:c.170_173del:p.57_58del	-	-	-	-	H17;H43	2	0
NYNRIN	nonframeshift deletion	NM_025081.3:c.171_173del:p.57_58del	-	-	-	-	H17	1	0
NYNRIN	frameshift deletion	NM_025081.3:c.5040delC:p.A1680fs	-	-	-	-	H18	1	0
NYNRIN	nonframeshift substitution	NM_025081.3:c.167_173AGGC	-	-	-	-	H45	1	0
NYNRIN	nonframeshift substitution	NM_025081.3:c.5038_5042GCCAG	-	-	-	-	H22	1	0
NYNRIN	frameshift substitution	NM_025081.3:c.167_173AGC	-	-	-	-	H45	1	0
NYNRIN	frameshift insertion	NM_025081.3:c.454_455insCG:p.R152fs	-	-	-	-	H29	1	0
NYNRIN	frameshift deletion	NM_025081.3:c.168delG:p.E56fs	-	-	-	-	H12	1	0
NYNRIN	frameshift substitution	NM_025081.3:c.5038_5042GCCA	-	-	-	-	H22	1	0
OPLAH	frameshift substitution	NM_017570.5:c.2189_2195GCCCCA	-	-	-	-	H17;H21;H40;H9	4	0
OPLAH	frameshift deletion	NM_017570.5:c.3668delA:p.N1223fs	-	-	-	-	H31;H9	2	0
OPLAH	nonsynonymous SNV	NM_017570.5::c.C2568T:p.H856=	-	-	rs377155890	Benign	H31	1	0
OSBPL7	frameshift deletion	NM_145798.3:c.651_652del:p.217_218del	-	-	-	-	H15;H22;H25;H46;H9	5	0
OSBPL7	nonframeshift substitution	NM_145798.3:c.348_352ACCCT	-	-	-	-	H29;H9	2	0
OSBPL7	frameshift substitution	NM_145798.3:c.348_352GCCT	-	-	-	-	H29;H9	2	0
PARP10	frameshift deletion	NM_032789.5:c.146delG:p.G49fs	-	-	-	-	H37;H48;H8;H9	4	0
PARP10	frameshift deletion	NM_032789.5:c.527delG:p.G176fs	-	-	-	-	H21	1	0
PARP10	frameshift substitution	NM_032789.5:c.16_20ACCA	-	-	-	-	H33	1	0
PARP10	frameshift deletion	NM_032789.5:c.2338delC:p.R780fs	-	-	-	-	H35	1	0
PARP10	frameshift deletion	NM_032789.5:c.3053delT:p.L1018fs	-	-	-	-	H17	1	0
PARP10	frameshift deletion	NM_032789.5:c.2634delG:p.R878fs	-	-	-	-	H40	1	0
PLCG1	nonsynonymous SNV	NM_002660.3:c.G3473A:p.R1158H	Deleterious	Damaging	rs141684852	-	H3	1	0

PLCG1	frameshift deletion	NM_002660.3:c.561delG:p.R187fs	-	-	-	-	H43	1	0
PLCG1	nonsynonymous SNV	NM_002660.3:c.G805C:p.G269R	-	-	-	-	H33	1	0
PVRL2/ NECTIN2	nonsynonymous SNV	NM_002856.3:c.G526C:p.D176H	Deleterious	Possible Damaging	rs146534542	Benign	H12	1	0
PVRL2/ NECTIN2	frameshift deletion	NM_002856.3:c.1281delG:p.L427fs	-	-	-	-	H25	1	0
SHARPIN	nonsynonymous SNV	NM_030974.4:c.G1094A:p.R365H	Deleterious	Damaging	rs112552278	Benign		1	0
SHARPIN	frameshift substitution	NM_030974.4:c.610_614GCCT	-	-	-	-	H40	1	0
SORT1	nonframeshift substitution	NM_002959.7:c.594_595CA	-	-	-	-	H32	1	0
SORT1	frameshift deletion	NM_002959.7:c.594delT:p.F198fs	-	-	-	-	H32	1	0
SPATC1	nonsynonymous SNV	NM_198572.3:c.C1504A:p.Q502K	Deleterious	Damaging	rs201925917	-	H21;H39	2	0
SPATC1	frameshift deletion	NM_198572.3:c.330delG:p.P110fs	-	-	-	-	H32	1	0
SPATC1	frameshift deletion	NM_198572.3:c.525delG:p.V175fs	-	-	-	-	H31	1	0
SPATC1	frameshift deletion	NM_198572.3:c.744delG:p.V248fs	-	-	-	-	H22	1	0
SPATC1	nonframeshift substitution	NM_198572.3:c.329_330CA	-	-	-	-	H32	1	0
SPATC1	stopgain SNV	NM_198572.3:c.C1606T:p.R536X	Tolerated	-	rs373716422	-	H31	1	0
ST3GAL4	nonsynonymous SNV	NM_006278.3:c.C824T:p.A275V	-	-	-	-	H3	1	0
ST3GAL4	nonsynonymous SNV	NM_006278.3:c.G773A:p.G256D	-	-	-	-	H48	1	0
TOMM40	frameshift deletion	NM_001128917.2:c.240delG:p.P80fs	-	-	-	-	H33;H39	2	0
ZHX3	frameshift deletion	NM_001384315.1:c.1417delG:p.V473fs	-	-	-	-	H35	1	0
ZHX3	nonsynonymous SNV	NM_001384315.1:c.C1232G:p.P411R,	Tolerated	Damaging	-	-	H14	1	0

Abbreviations: LDL-C; low density lipoprotein, HLDL; high LDL-C group, LLDL; low LDL-C group.

Supplementary Table S6. Summary of the 56 causative variants in 54 of tier-3 genes identified from whole-exome sequencing.

Gene	Variant Type	Nucleotide and amino acid changes	SIFT prediction	Polyphen2 prediction	snp138	Clinical Significance (ClinVar)	Sample IDs	Frequency in HLDL, n	Frequency in LLDL, n
ACADVL	frameshift deletion	NM_000018.4:c.74delC:p.T25fs	-	-	-	-	H14;H17;H18;H21;H29;H31;H37;H39;H43;H45;H48	11	0
AIM1L/ CRYBG2	nonsynonymous SNV	NM_001039775.4:c.G1430A:p.R477Q	-	-	rs201245727	-	H14;H15;H17;H25;H31;H40;H43;H45;H8;H9	10	0
APOBEC3F	frameshift deletion	NM_145298.6:c.840delC:p.C280fs	-	-	-	-	H14;H15;H16;H18;H21;H25;H29;H2; H31;H35;H37;H43;H8;H9	14	0
LOC100289470	nonsynonymous SNV	NC_000005.10:g.179522780A>C	-	-	rs28420183	-	H15;H16;H17;H18;H21;H22;H31;H40;H46;H48;H8	11	0
C1orf85/ GLMP	frameshift deletion	NM_144580.3:c.35_36del:p.12_12del	-	-	-	-	H14;H15;H16;H17;H18;H22;H25;H29;H31; H32;H33;H37;H40;H45;H48;H8;H9	17	0
CACNA1S	frameshift deletion	NM_000069.3:c.1330delC:p.L444fs	-	-	-	-	H14;H15;H18;H21;H25;H29;H37;H39;H48;H9	10	0
CCDC108/CFAP65	frameshift deletion	NM_194302.4:c.977delG:p.G326fs	-	-	-	-	H17;H18;H22;H25;H29;H35;H40;H45;H8;H9	10	0
CCDC168	nonsynonymous SNV	NM_001146197.3:c.A8434C:p.T2812P	-	-	rs9585986	-	H15;H17;H21;H22;H25;H29;H33;H35 H40;H46;H48;H8;H9	13	0
CDH20	frameshift deletion	NM_031891.4:c.2073delG:p.A691fs	-	-	-	-	H14;H16;H18;H29;H33;H40;H43;H45;H46;H9	10	0
CDK2	frameshift deletion	NM_001798.5:c.662delC:p.T221fs	-	-	-	-	H21;H22;H25;H31;H32;H35;H39;H40;H43;H46	10	0
CNTNAP3B	nonsynonymous SNV	NM_001201380.3:c.T1717G:p.C573G	Deleterious	-	rs76032838	-	H16;H17;H21;H22;H29;H2;H32; H35;H37;H39;H40;H43;H45;H46	14	0
DDI1	frameshift deletion	NM_001001711.3:c.788delC:p.A263fs	-	-	-	-	H14;H16;H21;H25;H29;H31;H32; H37;H40;H43;H45;H48;H8;H9	14	0
DNAH7	frameshift deletion	NM_018897.3:c.9955_9956del:p.3319_3319del	-	-	-	-	H14;H17;H25;H29;H31;H37;H40;H43;H48;H8;H9	11	0
DPCR1/ MUCL3	frameshift deletion	NM_080870.4:c.2453delC:p.S818fs	-	-	-	-	H14;H15;H16;H17;H21;H22;H25;H29;H2 H31;H33;H35;H39;H45;H46;H9	16	0
EZH1	frameshift deletion	NM_001991.5:c.579delG:p.G193fs	-	-	-	-	H14;H16;H17;H22;H31;H37;H39;H40;H45;H48	10	0
FAM114A1	frameshift deletion	NM_138389.4:c.573delG:p.Q191fs	-	-	-	-	H17;H21;H25;H31;H32;H35;H39;H40;H46;H8;H9	11	0
FRG1BP	nonframeshift insertion	NR_145491.1:n.509TTG	-	-	rs112430454	-	H12;H15;H16;H17;H18;H21;H22; H25;H29;H35 H39;H40;H43;H45;H46;H48;H8;H9	18	0

GBP7	frameshift deletion	NM_207398.3:c.460delG:p.A154fs	-	-	-	-	H15;H16;H17;H18;H21;H31;H32;H35;H37 H39;H40;H43;H45;H48;H8;H9	16	0
GTF3C1	frameshift deletion	NM_001520.4:c.167delG:p.G56fs	-	-	-	-	H14;H17;H18;H21;H25;H32;H33;H37; H39;H40;H43;H45;H46;H48;H8	15	0
HEATR5A	frameshift deletion	NM_015473.4:c.2656delG:p.V886fs	-	-	-	-	H15;H16;H17;H18;H21;H22;H25;H29; H31;H32;H33;H39;H46;H8;H9	15	0
HEATR5A	nonframeshift substitution	NM_015473.4:c.2656_2657AT	-	-	-	-	H15;H16;H17;H18;H21;H22;H25;H29; H31;H32;H33;H39;H46;H8;H9	15	0
ISLR2	frameshift deletion	NM_001130136.1:c.1827delG:p.L609fs	-	-	-	-	H15;H17;H18;H21;H22;H25;H2;H31; H48;H8;H9	11	0
ITGAX	frameshift deletion	M_001286375.2:c.1271_1272del:p.424_424del	-	-	-	-	H14;H16;H17;H18;H21;H29;H2;H31; H33; H39;H40;H43;H9	13	0
KBTBD12	frameshift deletion	NM_207335.4:c.159delC:p.S53fs	-	-	-	-	H14;H15;H16;H18;H21;H22;H2;H31; H39;H48;H8	11	0
KCNQ3	stopgain SNV	NM_004519.4:c.1702delA:p.I568X	-	-	-	-	H14;H15;H18;H29;H31;H40;H43;H45;H48;H8;H9	11	0
KEAP1	frameshift deletion	NM_203500.2:c.966delC:p.P322fs	-	-	-	-	H14;H15;H16;H17;H18;H21;H22;H25;H2;H31;H32 H37;H39;H40;H43;H45;H48;H8	18	0
KIAA2018/ USF3	frameshift deletion	NM_001009899.4:c.4589delG:p.G1530fs	-	-	-	-	H14;H21;H22;H25;H29;H31;H32;H33;H37;H40	10	0
KMT2C	nonsynonymous SNV	NM_170606.3:c.C871T:p.L291F	Tolerated	Damaging	rs56850341	-	H15;H17;H22;H29;H35;H39;H40;H45;H46;H8;H9	11	0
KMT2D	frameshift deletion	NM_003482.4:c.8418delG:p.G2806fs	-	-	-	-	H14;H16;H17;H18;H25;H31;H33;H39;H40;H43;H46	11	0
LAMC3	frameshift deletion	NM_006059.4:c.4505delC:p.A1502fs	-	-	-	-	H14;H17;H18;H21;H22;H25;H37;H39; H40;H46;H8;H9	12	0
MICALL2	frameshift deletion	NM_182924.4:c.2260delC:p.L754fs	-	-	-	-	H14;H17;H21;H25;H29;H32;H37;H39;H45;H8;H9	11	0
MTUS2	frameshift deletion	NM_001033602.4:c.1730delC:p.S577fs	-	-	-	-	H15;H16;H17;H18;H21;H31;H32;H35;H39; H40;H45;H48;H8;H9	14	0
MUC12	nonsynonymous SNV	NM_001164462.2:c.A5251C:p.T1751P	Tolerated	-	rs71557212	-	H15;H16;H17;H21;H22;H25;H29;H31;H35 H37;H39;H40;H43;H45;H46;H8;H9	17	0

MUC4	frameshift deletion	NM_018406.7:c.13180delC:p.P4394fs	-	-	-	-	H14;H17;H18;H21;H25;H29;H32;H39;H40;H9	10	0
NLRP14	frameshift deletion	NM_176822.4:c.638delT:p.V213fs	-	-	-	-	H17;H21;H22;H31;H32;H33;H39;H3;H43;H45;H46	11	0
OVCH2	frameshift deletion	NM_198185.7:c.1239delG:p.G413fs	-	-	-	-	H21;H22;H29;H31;H32;H33;H35;H40;H43;H45;H48;H8;H9	13	0
PIGR	nonsynonymous SNV	NM_002644.4:c.G1093A:p.G365S	Tolerated	Damaging	rs2275531	-	H15;H17;H21;H29;H33;H39;H45;H46;H48;H9	10	0
PLD4	frameshift deletion	NM_001308174.2:c.53delC:p.A18fs	-	-	-	-	H14;H15;H16;H17;H31;H35;H39;H40;H43;H45;H46	11	0
PSD4	frameshift deletion	NM_012455.3:c.362delC:p.A121fs	-	-	-	-	H15;H18;H21;H22;H25;H35;H45;H48;H8;H9	10	0
PTPRCAP	frameshift deletion	NM_005608.3: c.419delC:p.P140fs	-	-	-	-	H17;H21;H22;H25;H29;H31;H32;H46;H48;H8;H9	11	0
PUF60	frameshift deletion	NM_078480.3:c.163delG:p.G55fs	-	-	-	-	H18;H21;H25;H32;H35;H40;H43;H46;H8;H9	10	0
RNF212	nonsynonymous SNV	NM_001193318.3:c.A784G:p.I262V	Tolerated	-	rs1670534	-	H14;H15;H16;H17;H18;H21;H25;H29;H31;H33;H35;H37;H40;H43;H8;H9	16	0
RNMT	frameshift substitution	NM_001308263.2:c.926_930GCCT	-	-	-	-	H17;H18;H22;H25;H33;H37;H39;H40;H46;H8;H9	11	0
RYR1	frameshift deletion	NM_000540.3:c.813delC:p.S271fs	-	-	-	-	H15;H16;H18;H21;H22;H25;H39;H40;H43;H9	10	0
SEC13	frameshift deletion	NM_183352.3:c.950delG:p.G317fs	-	-	-	-	H14;H15;H16;H17;H18;H21;H22;H29;H31;H32;H33;H35;H37;H40;H43;H46;H8;H9	18	0
SGCE	frameshift deletion	NM_001099401.2:c.783delA:p.K261fs	-	-	-	-	H18;H25;H31;H33;H39;H40;H43;H45;H46;H48;H8;H9	12	0
SH3D19	frameshift deletion	NM_001009555.4:c.555_556del:p.185_186del	-	-	-	-	H14;H15;H21;H31;H32;H35;H37;H40;H48;H8	10	0
SLC35F6	frameshift deletion	NM_017877.4:c.911delG:p.W304fs	-	-	-	-	H14;H15;H17;H29;H32;H35;H45;H46;H8;H9	10	0
SPATA21	stopgain SNV	NM_198546.1:c.790delC:p.L264X	-	-	-	-	H14;H18;H21;H25;H29;H33;H43;H48;H8;H9	10	0
TIAM2	nonsynonymous SNV	NM_012454.4:c.G995A:p.R332H	Tolerated	Damaging	rs931312	-	H15;H16;H17;H18;H21;H22;H25;H29;H33;H35;H37;H40;H45;H9	14	0

TIMELESS	frameshift deletion	NM_003920.5:c.3280delG:p.A1094fs	-	-	-	-	H14;H15;H17;H18;H21;H22;H29;H31 H32;H35;H39;H40;H45;H46;H8	15	0
TNR	frameshift deletion	NM_003285.3:c.1951delG:p.A651fs	-	-	-	-	H15;H17;H18;H21;H22;H25;H31;H39 ;H40;H9	10	0
UBR1	frameshift deletion	NM_174916.3:c.4519delC:p.P1507fs	-	-	-	-	H17;H18;H21;H22;H25;H31;H32;H33 ;H35 H37;H39;H40;H43;H46;H48;H8;H9	17	0
WDR74	frameshift deletion	NM_018093.3:c.855delC:p.A285fs	-	-	-	-	H14;H15;H16;H17;H18;H21;H22;H25 ;H31;H32 H37;H39;H40;H43;H45;H46;H48;H8; H9	19	0
WDR90	frameshift deletion	NM_145294.5:c.4701delG:p.Q1567fs	-	-	-	-	H14;H15;H17;H21;H29;H31;H32 H37;H39;H40;H43;H48;H8;H9	14	0
ZNF687	frameshift deletion	NM_001304763.2:c.3delG:p.M1fs	-	-	-	-	H14;H18;H21;H22;H25;H29;H33 H35;H37;H40;H46;H48;H8	13	0

Abbreviations: LDL-C; low density lipoprotein, HLDL; high LDL-C group, LLDL; low LDL-C group.

Supplementary Table S7. Summary of the 17 protective variants in tier-1 genes identified from whole-exome sequencing.

Gene	Variant Type	Nucleotide and amino acid changes	SIFT prediction	Polyphen2 prediction	snp138	Clinical Significance (ClinVar)	Sample IDs	Frequency in HLDL, n	Frequency in LLDL, n
APOB	nonsynonymous SNV	NM_000384.3:c.G7331A:p.R2444H	Tolerated	Benign	rs200143030	Conflicting pathogenicity	L49;L4	0	2
APOB	nonsynonymous SNV	NM_000384.3:c.G4163A:p.R1388H	Tolerated	Benign	rs13306187	Conflicting pathogenicity	L49;L4	0	2
APOB	nonsynonymous SNV	NM_000384.3:c.G10913A:p.R3638Q	Tolerated	Benign	rs1801701	-	L19	0	1
APOB	stopgain SNV	NM_000384.3:c.T387A:p.Y129X	Deleterious	-	-	-	L24	0	1
APOB	nonsynonymous SNV	NM_000384.3:c.C4274G:p.S1425C	Deleterious	Possible Damaging	-	-	L38	0	1
APOB	synonymous SNV	NM_000384.3:c.C5430T:p.N1810=	-	-	rs1352777306	-	L4	0	1
APOB	synonymous SNV	NM_000384.3:c.C3219T:p.L1073=	-	-	rs750588820	Likely benign	L11	0	1
APOB	stopgain SNV	NM_000384.3:c.C8240G:p.S2747X	Deleterious	-	-	-	L11	0	1
APOB	stopgain SNV	NM_000384.3:c.T5340G:p.Y1780X	Tolerated	-	-	-	L49	0	1
LDLR	synonymous SNV	NM_000527.5:c.C1725T:p.L575=	-	-	rs1799898	Benign/Likely benign	L27;L45	0	2
LDLRAP1	nonframeshift substitution	NM_015627.3:c.603_604CC	-	-	-	-	L46	0	1
PCSK9	nonsynonymous SNV	NM_174936.4:c.C277T:p.R93C	Tolerated	Damaging	rs151193009	Conflicting pathogenicity	L13;L19;L40	0	3
PCSK9	frameshift deletion	NM_174936.4:c.83delC:p.A28fs	-	-	-	-	L11;L27	0	2
PCSK9	nonframeshift insertion	NM_174936.4:c.42_43insCTG:p.P14delinsPL	-	-	-	-	L28;L42	0	2
PCSK9	synonymous SNV	NM_174936.4:c.C141T:p.S47=	-	-	rs28385701	Conflicting pathogenicity	L8	0	1
PCSK9	synonymous SNV	NM_174936.4:c.C993T:p.P331=	-	-	rs376753957	Benign/Likely benign	L38	0	1
PCSK9	synonymous SNV	NM_174936.4: c.C351T:p.G117=	-	-	-	-	L25	0	1

Abbreviations: LDL-C; low density lipoprotein, HLDL; high LDL-C group, LLDL; low LDL-C group.

Supplementary Table S8. Summary of the 62 protective variants in 10 tier-2 genes identified from whole-exome sequencing.

Gene	Variant Type	Nucleotide and amino acid change	SIFT prediction	Polyphen2 prediction	snp138	Clinical Significance (ClinVar)	Sample IDs	Frequency in HLDL, n	Frequency in LLDL, n
CELSR2	frameshift deletion	NM_001408.3:c.1505delC:p.A502fs	-	-	-	-	L38;L8	0	2
CELSR2	nonsynonymous SNV	NM_001408.3:c.G6340A:p.A2114T	Tolerated	Possible Damaging	rs375166359	-	L13	0	1
CELSR2	nonsynonymous SNV	NM_001408.3:c.G8642C:p.R2881P	Tolerated	Benign	rs761753467	-	L27	0	1
CELSR2	frameshift deletion	NM_001408.3:c.3524delC:p.A1175fs	-	-	-	-	L28	0	1
CELSR2	frameshift deletion	NM_001408.3:c.3528delC>T:p.P1176fs	-	-	-	-	L14	0	1
CELSR2	synonymous SNV	NM_001408.3:c.C3534G:p.G1178G	-	-	-	-	L49	0	1
CELSR2	frameshift deletion	NM_001408.3:c.8086delC:p.P2696fs	-	-	-	-	L24	0	1
CELSR2	synonymous SNV	NM_001408.3:c.G5364A:p.E1788E	-	-	rs767874482	-	L45	0	1
CELSR2	synonymous SNV	NM_001408.3:c.A8628G:p.G2876G	-	-	rs441853	-	L39	0	1
CELSR2	nonsynonymous SNV	NM_001408.3:c.C2084T:p.T695M	Tolerated	Damaging	rs117469174	-	L25	0	1
CELSR2	nonsynonymous SNV	NM_001408.3:c.G425T:p.R142L	Tolerated	Benign	-	-	L49	0	1
CELSR2	frameshift deletion	NM_001408.3:c.8085_8086del:p.2695_2696 del	-	-	-	-	L49	0	1
CELSR2	nonsynonymous SNV	NM_001408.3:c.C8417T:p.A2806V	Tolerated	Benign	rs77619489	-	L42	0	1
CELSR2	synonymous SNV	NM_001408.3:c.G780A:p.T260T	-	-	rs372864529	-	L13	0	1
CELSR2	nonsynonymous SNV	NM_001408.3: c.5134C>AG: p. P1712A	Tolerated	Benign	rs1455877280	-	L25	0	1
CELSR2	nonsynonymous SNV	NM_001408.3:c.G1792A:p.V598M	Deleterious	Benign	rs117684956	-	L25	0	1
DCPS	synonymous SNV	NM_014026.6:c.G558A:p.A186A	-	-	rs188471699	Benign	L42	0	1
DCPS	nonsynonymous SNV	NM_014026.6:c.C947T:p.T316M	Deleterious	Possible Damaging	rs137941190	Pathogenic/ Likely pathogenic	L43	0	1
DCPS	nonframeshift insertion	NM_014026.6:c.215_216insTGGGGA:p.S72 delinsSGD	-	-	rs201095573	-	L26	0	1
GPAA1	frameshift deletion	NM_003801.4:c.739delC:p.P247fs	-	-	-	-	L23	0	1
LPA	frameshift deletion	NM_005577.4:c.3148delG:p.G1050fs	-	-	-	-	L23;L39;L49;L8	0	4
LPA	frameshift insertion	NM_005577.4:c.3147dupC:p.G1050fs	-	-	-	-	L23;L39;L49;L8	0	4

LPA	nonsynonymous SNV	NM_005577.4:c.A5673G:p.I1891M	Deleterious	Damaging	rs3798220	Benign	L14;L26;L35	0	3
LPA	synonymous SNV	NM_005577.4:c.C2826T:p.H942H	-	-	rs117174672	-	L26;L39	0	2
LPA	nonsynonymous SNV	NM_005577.4:c.C2945T:p.A982V	-	Damaging	rs139937718	-	L25	0	1
MAF1	frameshift deletion	NM_032272.5:c.715delG:p.G239fs	-	-	-	-	L46	0	1
NYNRIN	frameshift substitution	NM_025081.3:c.167_172AGGGC	-	-	-	-	L14;L19;L26;L38;L39;L46;L8	0	7
NYNRIN	frameshift deletion	NM_025081.3:c.2247delG:p.E749fs	-	-	-	-	L28;L43;L50	0	3
NYNRIN	frameshift deletion	NM_025081.3:c.168_169del:p.56_57del	-	-	-	-	L43;L50	0	2
NYNRIN	frameshift deletion	NM_025081.3:c.169delG:p.G57fs	-	-	-	-	L43;L50	0	2
NYNRIN	frameshift deletion	NM_025081.3:c.2249delG:p.G750fs	-	-	-	-	L13;L27	0	2
NYNRIN	frameshift deletion	NM_025081.3:c.1216_1217del:p.406_406del	-	-	-	-	L2	0	1
NYNRIN	frameshift deletion	NM_025081.3:c.3338delC:p.A1113fs	-	-	-	-	L40	0	1
NYNRIN	synonymous SNV	NM_025081.3:c.G5445A:p.R1815=	-	-	rs1355473794	-	L26	0	1
NYNRIN	synonymous SNV	NM_025081.3:c.G4590A:p.K1530K	-	-	rs368816036		L45	0	1
NYNRIN	nonframeshift deletion	NM_025081.3:c.2247_2249del:p.749_750del	-	-	-	-	L13	0	1
NYNRIN	frameshift deletion	NM_025081.3:c.143delC:p.T48fs	-	-	-	-	L40	0	1
NYNRIN	nonsynonymous SNV	NM_025081.3:c.T4946C:p.F1649S	Deleterious	Damaging	-	-	L24	0	1
NYNRIN	synonymous SNV	NM_025081.3:c.G1485A:p.P495P	-	-	rs12897153	-	L14	0	1
NYNRIN	frameshift deletion	NM_025081.3:c.1217delG:p.G406fs	-	-	-	-	L2	0	1
NYNRIN	nonsynonymous SNV	NM_025081.3:c.C3320G:p.A1107G	Tolerated	Benign	-	-	L11	0	1
OPLAH	synonymous SNV	NM_017570.5:c.C1989T:p.T663=	-	-	-	-	L40;L45	0	2
OPLAH	frameshift deletion	NM_017570.5:c.2608delC:p.H870fs	-	-	-	-	L49	0	1
OPLAH	nonsynonymous SNV	NM_017570.5:c.G1973T:p.R658L	Tolerated	Benign	rs148181675	Benign	L14	0	1

OPLAH	nonsynonymous SNV	NM_017570.5:c.G364A:p.A122T	Tolerated	Damaging	rs199914741	-	L46	0	1
OPLAH	synonymous SNV	NM_017570.5:c.C195T:p.L65L	-	-	-	-	L9	0	1
OPLAH	nonsynonymous SNV	NM_017570.5:c.C1793G:p.A598G	Tolerated	Benign	-	-	L40	0	1
OSBPL7	frameshift substitution	NM_145798.3:c.1185_1189TGGA	-	-	-	-	L4	0	1
OSBPL7	nonframeshift substitution	NM_145798.3:c.1185_1189CGGGA	-	-	-	-	L4	0	1
OSBPL7	synonymous SNV	NM_145798.3:c.C189T:p.N63N	-	-	rs140138342	-	L2	0	1
PARP10	frameshift deletion	NM_032789.5:c.538delG:p.D180fs	-	-	-	-	L27;L43	0	2
PARP10	synonymous SNV	NM_032789.5:c.G2214A:p.T738=	-	-	rs370075141	-	L8	0	1
PARP10	frameshift deletion	NM_032789.5:c.2096delC:p.P699fs	-	-	-	-	L49	0	1
PARP10	frameshift deletion	NM_032789.5:c.2011delG:p.A671fs	-	-	-	-	L24	0	1
PARP10	nonsynonymous SNV	NM_032789.5:c.G3059C:p.G1020A	Tolerated	Benign	-	-	L26	0	1
PARP10	nonframeshift substitution	NM_032789.5:c.3050_3052CTC	-	-	-	-	L10	0	1
PARP10	frameshift deletion	NM_032789.5:c.3050_3051del:p.1017_1017 del	-	-	-	-	L10	0	1
SPATC1	frameshift deletion	NM_198572.3:c.785delC:p.P262fs	-	-	-	-	L24;L8	0	2
SPATC1	nonsynonymous SNV	NM_198572.3:c.T193C:p.S65P	Tolerated	Benign	rs60050811	-	L26;L46	0	2
SPATC1	nonsynonymous SNV	NM_198572.3:c.C785T:p.P262L	Tolerated	Benign	rs145229001	-	L19	0	1
SPATC1	nonsynonymous SNV	NM_198572.3:c.G1651A:p.V551M	Tolerated	Benign	rs370797939	-	L40	0	1
SPATC1	synonymous SNV	NM_198572.3:c.C288G:p.P96P	-	-	rs374114772	-	L27	0	1

Abbreviations: LDL-C; low density lipoprotein, HLDL; high LDL-C group, LLDL; low LDL-C group.

Supplementary Table S9. Summary of the 23 protective variants in 23 tier-3 genes identified from whole-exome sequencing.

Gene	Variant Type	Nucleotide and amino acid change	SIFT prediction	Polyphen2 prediction	snp138	Clinical Significance (ClinVar)	Sample IDs	Frequency in HLDL, n	Frequency in LLDL, n
ACIN1	frameshift deletion	NM_014977.3:c.2411delG:p.G804fs	-	-	-	-	L11;L27;L2;L38;L40;L46;L49;L4;L50;L8	0	10
AGAP1	frameshift deletion	NM_001037131.3:c.380_381del:p.127_127del	-	-	-	-	L19;L24;L26;L27;L2;L42;L45;L46;L4;L8	0	10
BMP1	frameshift deletion	NM_006129.5:c.2442delG:p.K814fs	-	-	-	-	L11;L13;L19;L27;L28;L35;L40;L42;L43;L45;L49;L4;L50	0	13
CASC3	frameshift deletion	NM_007359.5:c.173delC:p.A58fs	-	-	-	-	L23;L25;L26;L27;L28;L2;L35;L38;L43;L45;L4;L50;L8	0	13
CPEB3	frameshift deletion	NM_014912.5:c.258delT:p.P86fs	-	-	-	-	L11;L13;L14;L19;L26;L27;L28;L2;L42;L8	0	10
CPSF1	nonsynonymous SNV	NM_013291.3:c.937+22C>T	-	-	rs141140965	-	L10;L11;L13;L24;L2;L35;L42;L43;L45;L46	0	10
CYP4F8	frameshift deletion	NM_007253.4:c.555delC:p.G185fs	-	-	-	-	L14;L26;L27;L2;L35;L38;L42;L43;L50;L8	0	10
EWSR1	frameshift substitution	NM_013986.4:c.1286_1289AGC	-	-	-	-	L19;L24;L28;L2;L35;L38;L42;L43;L4;L50;L9	0	11
HNF1A	frameshift deletion	NM_001306179.2:c.1124_1125del:p.375_375del	-	-	-	-	L14;L19;L26;L2;L35;L38;L42;L43;L45;L46;L50	0	11
IGSF3	nonsynonymous SNV	NM_001542.4:c.C1345T:p.R449C	Deleterious	Damaging	rs968366784	-	L10;L13;L14;L24;L27;L39;L40;L42;L43;L50	0	10
KCP	frameshift deletion	NM_199349.3:c.46delG:p.A16fs	-	-	rs11335250	-	L11;L13;L19;L24;L26;L27;L28;L39;L42;L43;L49	0	11
KHSRP	frameshift deletion	NM_003685.3:c.1122_1123del:p.374_375del	-	-	-	-	L19;L24;L28;L2;L35;L38;L43;L46;L50;L8	0	10
KIDINS220	frameshift deletion	NM_020738.4:c.4199delG:p.G1400fs	-	-	-	-	L19;L24;L26;L28;L2;L35;L38;L42;L45;L8	0	10
MAP7D2	frameshift deletion	NM_001168465.2:c.2010delC:p.A670fs	-	-	-	-	L19;L23;L27;L28;L35;L38;L43;L50;L8;L9	0	10
MOCS2	frameshift deletion	NM_176806.4:c.252delC:p.P22fs	-	-	-	-	L13;L26;L28;L2;L35;L38;L42;L43;L46;L8;L9	0	11

MYO7B	frameshift deletion	NM_001080527.2:c.5331delG:p.T1777fs	-	-	-	-	L13;L14;L19;L27;L28;L2;L38retry;L42;L43;L4;L50;L9	0	12
OTOP1	nonframeshift deletion	NM_177998.3:c.310_318del:p.104_106del	-	-	rs111245977	-	L13;L14;L26;L27;L28;L35;L38;L39;L43;L45;L46;L50;L9	0	13
SHANK1	frameshift deletion	NM_016148.5:c.1714delG:p.E572fs	-	-	-	-	L10;L13;L14;L19;L26;L27;L28;L35;L38;L39;L42;L43;L45;L50;L8	0	15
SIPA1	frameshift deletion	NM_153253.30:c.856delC:p.P286fs	-	-	-	-	L19;L26;L27;L28;L2;L35;L38;L39;L42;L43;L45;L46;L4;L50;L8;L9	0	16
SLC2A13	frameshift deletion	NM_052885.4:c.469delC:p.L157fs	-	-	-	-	L10;L19;L25;L26;L27;L28;L38;L39;L42;L43;L45;L46;L49;L50;L8	0	15
SLC2A4RG	frameshift deletion	NM_020062.4:c.48delG:p.L16fs	-	-	-	-	L19;L24;L26;L28;L2;L35;L38retry;L42;L46;L50;L8;L9	0	12
SNX32	frameshift deletion	NM_152760.3:c.141+64delG	-	-	rs11327250	-	L13;L14;L26;L27;L2;L35;L38;L42;L43;L45;L50;L9	0	12
TAS2R19	stoploss SNV	NM_176888.2:c.A900G:p.X300W	-	-	rs79475879	-	L11;L13;L19;L23;L24;L28;L35;L38;L39;L42;L43	0	11

* **Abbreviations:** LDL-C; low density lipoprotein, HLDL; high LDL-C group, LLDL; low LDL-C group.

Supplementary Table S10. Multiple logistic regression models of non-genetic risk factors associated with HLDL in males and females.

**Non-genetic Risk Factor**	**Male**		**Female**	
	**aOR (95%CI)**	**p-value**	**aOR (95%CI)**	**p-value**
Age (years)	NIL		1·07 (1·03, 1·10)	<0·001*
Fasting blood glucose (mmol/L)	NIL		1·23 (1·12, 1·36)	<0·001*
Ever-use of tobacco products (Yes)	1.90 (1·06, 3.37)	0·030*	NIL	
Diabetes with medication status (Yes)	0·43 (0·24, 0·79)	0·006*	0·17 (0·08, 0·36)	<0·001*
Family history of hyperlipidemia (Yes)	NIL		NIL	
Hyperlipidemia with medication (Yes)	NIL		3.58 (1.99, 6.45)	<0.001*
Nagelkerke's R2	0·07		0·14	
AUC (95%CI)	0·61 (0·54, 0·69)		0·69 (0·65, 0·74)	

*** p-value < 0.05, Abbreviations:** aOR, adjusted odds ratio; 95%CI, 95% confidence interval; AUC, area under the curve.

Supplementary Table S11. Multiple logistic regression models of genetic risk factors associated with HLDL in males and females.

**Genetic Risk Factor**	**Male**		**Female**	
	**aOR (95%CI)**	**p-value**	**aOR (95%CI)**	**p-value**
*T2FH_OSBPL7*_01	14·9 (4·23, 52·42)	<0·001*	32·0 (3·18, 322·1)	0·003*
*T2FH_SPATC1*_01	0·20 (0·05, 0·78)	0·02*	NIL	
rs151193009	0·12 (0·02,0·57)	0·008*	0·05 (0·003, 0·68)	0·03*
Nagelkerke's R^2	0·17		0·17	
AUC (95%CI)	0·61 (0·54, 0·68)		0·60 (0·55, 0·66)	

*** p-value < 0.05, Abbreviations:** aOR, adjusted odds ratio; 95%CI, 95% confidence interval; AUC, area under the curve.
